# Complete Genome Sequences of 38 *Gordonia* sp. Bacteriophages

**DOI:** 10.1128/genomeA.01143-16

**Published:** 2017-01-05

**Authors:** Welkin H. Pope, Matthew T. Montgomery, J. Alfred Bonilla, Randall Dejong, Rebecca A. Garlena, Carlos Guerrero Bustamante, Karen K. Klyczek, Daniel A. Russell, John T. Wertz, Deborah Jacobs-Sera, Graham F. Hatfull

**Affiliations:** a Department of Biological Sciences, University of Pittsburgh, Pittsburgh, Pennsylvania, USA; b Department of Biology, University of Wisconsin – River Falls, River Falls, Wisconsin, USA; c Department of Biology, Calvin College, Grand Rapids, Michigan, USA

## Abstract

We report here the genome sequences of 38 newly isolated bacteriophages using *Gordonia terrae* 3612 (ATCC 25594) and *Gordonia neofelifaecis* NRRL59395 as bacterial hosts. All of the phages are double-stranded DNA (dsDNA) tail phages with siphoviral morphologies, with genome sizes ranging from 17,118 bp to 93,843 bp and spanning considerable nucleotide sequence diversity.

## GENOME ANNOUNCEMENT

The bacteriophage population is vast, dynamic, and old, with an estimated population of 10^31^ virions and 10^23^ productive infections/s on a global scale (1). The genomic diversity of the population is poorly understood, with fewer than 3,000 complete genome sequences in GenBank. In general, phages isolated on phylogenetically unrelated hosts share little or no sequence similarity, but considerable insights can be gleaned by comparative genomics of phages isolated on a common host, as illustrated for enterobacteriophages and mycobacteriophages (2, 3). The Howard Hughes Medical Institute (HHMI) Science Education Alliance-Phage Hunters Advancing Genomics and Evolutionary Science (SEA-PHAGES) program provides an undergraduate course-based research experience that contributes to our understanding of phage diversity and evolution through bacteriophage discovery and genomics, using *Actinobacteria*, including mycobacteria and *Gordonia* sp. strains, as isolation hosts.

*Gordonia* phages were isolated by enrichment or direct plating of filtered soil samples using *Gordonia terrae* 3612 or *Gordonia neofelifaecis* NRRL 59395 as a host (Table 1). Thirty-eight individual phages were isolated, and electron microscopy shows that all have siphoviridal morphotypes. Plaque-purified phages were amplified, and their double-stranded DNA (dsDNA) was extracted and sequenced using an Illumina MiSeq, as described previously (4). The 140-base reads were assembled using Newbler and Consed, with average coverages between 447- and 3,241-fold. Sequence ambiguities and genome termini were resolved either by sequencing directly from genomic templates or from PCR products. Genomes were annotated using DNA Master (http://cobamide2.bio.pitt.edu), coding sequences were predicted using GeneMark (5) and Glimmer (6), and tRNAs were predicted using Aragorn (7) and tRNAscan-SE (8). Functional assignments were made using BLASTP (9) and HHpred (10, 11) against the publically available databases GenBank, the Protein Data Bank, and Pfam.

**TABLE 1:** *Gordonia* phage genometrics

Phage name	GenBank accession no.	Genome size (bp)	G+C content (%)	No. of tRNAs	No. of CDSs^a^	End type^b^	Host strain
Bachita^c^	KU998247	93,843	61.9	8	182	CGCGACGCTC	*G. terrae* 3612
Bantam^d^	KX557272	92,580	64.7	2	168	CGCAGCACTC	*G. terrae* 3612
BatStarr^e^	KX557273	53,432	66.6	0	83	CGGCTGGGGA	*G. terrae* 3612
Blueberry^c^	KU998236	54,990	67	0	86	TGGCCGGTGA	*G. terrae* 3612
BritBrat^c^	KU998233	55,524	65	0	98	CGTATGGCAT	*G. terrae* 3612
CaptainKirk2^e^	KX557274	47,898	67.4	0	79	TCGCCGGTGA	*G. terrae* 3612
CarolAnn^e^	KX557275	54,167	66.9	0	80	TGGCCGGTGA	*G. terrae* 3612
ClubL^c^	KU998246	92,618	61.9	9	179	CGCGACGCTC	*G. terrae* 3612
Cozz^c^	KU998239	46,600	60	0	68	CGGTAGGCTT	*G. terrae* 3612
Cucurbita^f^	KX557276	93,686	62	9	178	CGCGACGCTC	*G. terrae* 3612
Demosthenes^c^	KU998242	74,073	59.3	0	95	Dir. Term. Repeat	*G. terrae* 3612
Eyre^e^	KX557277	44,929	67.5	0	74	CCCTGCGCTGA	*G. terrae* 3612
Ghobes^e^	KX557278	45,285	65.2	0	59	TGCCCGAGGTA	*G. terrae* 3612
Hedwig^e^	KX557279	44,536	67.2	0	70	TCCCGCGGTA	*G. terrae* 3612
Howe^c^	KU252585	53,182	65.6	0	79	TGCCAAGGGGA	*G. terrae* 3612
JSwag^d^	KX557280	52,726	61.9	3	101	CGGGTGGTTA	*G. terrae* 3612
Jumbo^d^	KX557281	78,302	54.5	0	102	Dir. Term. Repeat	*G. terrae* 3612
Kampe^c^	KU998254	80,649	47	2^g^	115	Dir. Term. Repeat	*G. terrae* 3612
KatherineG^c^	KU998251	52,689	61.9	3	99	CGGGTGGTTA	*G. terrae* 3612
Kvothe^c^	KU998243	75,462	59.5	0	99	Dir. Term. Repeat	*G. terrae* 3612
Nyceirae^e^	KX557282	41,857	67.5	0	61	CGCGGGGGA	*G. terrae* 3612
OneUp^c^	KU998245	93,577	61.5	9	163	CGCGACGCTC	*G. terrae* 3612
Orchid^c^	KU998253	80,650	47	2^g^	114	Dir. Term. Repeat	*G. terrae* 3612
PatrickStar^c^	KU998252	80,729	47	2^g^	115	Dir. Term. Repeat	*G. terrae* 3612
Remus^c^	KX557283	52,738	62	3	98	CGGGTGGTTA	*G. terrae* 3612
Rosalind^c^	KU998250	52,684	61.9	3	99	CGGGTGGTTA	*G. terrae* 3612
Smoothie^c^	KU998244	93,139	61.9	8	179	CGCGACGCTC	*G. terrae* 3612
Soups^c^	KU998249	52,924	61.9	3	98	CGGGTGGTTA	*G. terrae* 3612
Splinter^c^	KU998238	45,858	66.1	0	80	TCCGGGCCGGTA	*G. terrae* 3612
Strosahl^d^	KX557284	52,738	62	3	98	CGGGTGGTTA	*G. terrae* 3612
Terrapin^e^	KX557285	66,611	59.6	0	97	Circ. Permuted	*G. terrae* 3612
Twister6^e^	KX557286	57,804	67.7	0	93	Circ. Permuted	*G. terrae* 3612
Utz^c^	KU998248	49,768	67.7	0	71	TCGCCGGTGA	*G. terrae* 3612
Vendetta^c^	KU998237	45,858	66.1	0	81	TCCGGGCCGGTA	*G. terrae* 3612
Wizard^c^	KU998234	58,308	67.9	0	89	Circ. Permuted	*G. terrae* 3612
Zirinka^e^	KX557287	52,077	66.7	0	79	CGGCTGGGGA	*G. terrae* 3612
Jeanie^c^	KU998256	17,118	68.6	0	25	AGCCCCCGGT	*G. neofelifaecis*
McGonagall^c^	KU998255	17,119	68.6	0	25	AGCCCCCGGT	*G. neofelifaecis*

a CDSs, coding sequences.

b End types are 3′-single-stranded overhangs, unless otherwise noted as Dir. Term. Repeat (direct terminal repeat) or Circ. Permuted (circularly permuted).

c Phage Hunters Integrating Research and Education (PHIRE) program, University of Pittsburgh.

d Science Education Alliance-Phage Hunters Advancing Genomics and Evolutionary Science (SEA-PHAGES), University of Wisconsin-River Falls.

e SEA-PHAGES, University of Pittsburgh.

f SEA-PHAGES, Calvin College.

g This total includes one transfer-messenger RNA (tmRNA).

The 38 newly isolated *Gordonia* phages exhibit considerable diversity (Table 1). The smallest genomes, Jeanie and McGonagall, at ~17,000 bp, have the highest G+C content (68%) and are each predicted to contain only 25 genes, including those encoding structural proteins, integrase and immunity repressor, endolysin, and a DnaQ-like subunit of DNA polymerase III. Three phages (PatrickStar, Kampe, and Orchid) have G+C contents (47%) that are strikingly lower than that of their host (67.77%), and lower than the G+C% of any mycobacteriophage; these phages may be relatively recent arrivals to the *Gordonia* neighborhood (12) (Table 1). These phages, together with Kvothe, Jumbo, and Demosthenes, have genomes with direct terminal repeats, a feature not observed in any mycobacteriophages. Many of the *Gordonia* phage genomes have defined ends with 3′ single-stranded extensions (Table 1), and only three (Terapin, Twister6, and Wizard) are circularly permuted.

Most of the *Gordonia* phages form turbid plaques, and 27 of the 38 encode either tyrosine or serine integrases; another six phages encode putative ParAB partitioning systems. Temperate lifestyles thus appear to be common for these phages. Some of the phages have all or part of a second integrase gene, and although these are mostly predicted to be nonfunctional, they perhaps reflect relatively recent genomic rearrangements. Finally, we note that six phages, KatherineG, Rosalind, Strosahl, Remus, Soups, and JSwag, are sufficiently similar to some mycobacteriophages to warrant grouping within Cluster A (13).

### Accession number(s).

Nucleotide sequence accession numbers are shown in Table 1.
